# Laser-induced thermal response and controlled release of copper oxide nanoparticles from multifunctional polymeric nanocarriers

**DOI:** 10.1080/14686996.2021.1883406

**Published:** 2021-03-12

**Authors:** Inbal Maor, Somayeh Asadi, Sanzhar Korganbayev, Daniel Dahis, Yosi Shamay, Emiliano Schena, Haim Azhari, Paola Saccomandi, Iris Sonia Weitz

**Affiliations:** a Department of Biotechnology Engineering, ORT Braude College, Karmiel, Israel; b Department of Mechanical Engineering, Politecnico di Milano, Milano, Italy; c Department of Biomedical Engineering, Technion–Israel Institute of Technology, Technion City, Israel; d Laboratory of Measurement and Biomedical Instrumentation, Università Campus Bio‐Medico di Roma, Rome, Italy

**Keywords:** CuO nanoparticles, PLGA, polydopamine, stimuli-responsive release, laser, photothermal therapy, multifunctional nanocarrier, light-absorbing material, 100 Materials; 102 Porous / Nanoporous / Nanostructured materials

## Abstract

Multifunctional nanocarriers have attracted considerable interest in improving cancer treatment outcomes. Poly(lactide-co-glycolide) (PLGA) nanospheres encapsulating copper oxide nanoparticles (CuO-NPs) are characterized by antitumor activity and exhibit dual-modal contrast-enhancing capabilities. An in vitro evaluation demonstrates that this delivery system allows controlled and sustained release of CuO-NPs. To achieve localized release on demand, an external stimulation by laser irradiation is suggested. Furthermore, to enable simultaneous complementary photothermal therapy, polydopamine (PDA) coating for augmented laser absorption is proposed. To this aim, two formulations of CuO-NPs loaded nanospheres are prepared from PLGA polymers RG-504 H (H-PLGA) and RG-502 H (L-PLGA) as scaffolds for surface modification through in situ polymerization of dopamine and then PEGylation. The obtained CuO-NPs-based multifunctional nanocarriers are characterized, and photothermal effects are examined as a function of wavelength and time. The results show that 808 nm laser irradiation of the coated nanospheres yields maximal temperature elevation (T = 41°C) and stimulates copper release at a much faster rate compared to non-irradiated formulations. Laser-triggered CuO-NP release is mainly depended on the PLGA core, resulting in faster release with L-PLGA, which also yielded potent anti-tumor efficacy in head and neck cancer cell line (Cal-33). In conclusion, the suggested multifunctional nanoplatform offers the integrated benefits of diagnostic imaging and laser-induced drug release combined with thermal therapy.

## GRAPHICAL ABSTRACT

**Figure:**
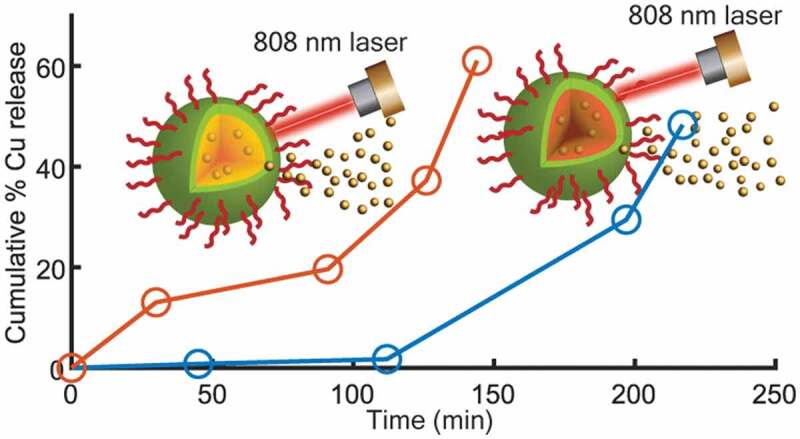

## 1. Introduction

Nanocarriers have been extensively studied to address aspects of anticancer multifunctional agents [1]. The building blocks of such materials are often made by integration and mixture of different components, which, as a whole, contribute to a variety of therapeutic and diagnostic capabilities [2]. However, due to the mono-functionality of most conventional agents, multi-component nanocarriers face difficulties in passing regulatory and safety requirements. To overcome these limitations, significant efforts have been devoted to seeking components possessing inherent multifunctional capabilities.

Copper oxide nanoparticles (CuO-NPs) are superior candidates for biomedical applications [3]. These NPs can serve as a contrast-enhancing material for two independent diagnostic imaging modalities: magnetic resonance imaging (MRI) and ultrasound [4]. CuO-NPs are among the most widely applied inorganic nano-materials, with remarkable antimicrobial activity against gram-positive and gram-negative bacterial strains [5–7]. These NPs are also a potentially useful agent to inhibit candidiasis [8] and viral infections [9] in human environments. Additionally, CuO-NPs display anticancer potencies in treating several cell lines (e.g. PANC1, HepG2, K562, and MCF7) [10]. Furthermore, an *in vivo* study showed that folate-conjugated CuO-NPs efficiently reduced the tumor volume of Dalton lymphoma and increased tumor cell death [11]. Recently, it was demonstrated how CuO-NPs inhibit the tumor growth of pancreatic tumors in mice models, partially by targeting tumor-initiating cells (TICs) [12], which are suggested to be responsible for cancer reoccurrence. Mechanistic studies proposed that CuO-NPs’ cytotoxicity is mediated by their ability to generate reactive oxygen species (ROS) [13] and disruption of mitochondrial functions [14]. As a consequence, during development of diagnostic and therapeutic purposes, concerns have been raised regarding the toxicity of CuO-NPs in high doses. To address this challenge, encapsulation of CuO-NPs in nanospheres (NS) composed of poly D,L-lactide-co-glycolide (PLGA, molecular weight (MW) 38–54 kDa, Resomer® RG 504 H) has been proposed as a delivery system exhibiting low toxicity towards pancreatic cancer cell lines (PANC-1) [15]. It was also shown that this nanocarrier preserved the CuO-NPs’ ultrasound imaging capability [15] and gave an even higher MRI performance compared to the bare CuO-NPs [16]. These PLGA nanospheres promote controlled and sustained release of CuO-NPs over a period of 36 days. Analysis of the *in vitro* data suggested their potential selective accumulation in tumor tissue through the enhanced permeability and retention (EPR) effect of leaky tumor vasculature [17]. Considering all the above, CuO-NPs are an attractive therapeutic drug and it will be beneficial for an effective cancer treatment to add stimuli-responsive features providing controlled biodistribution in tumor microenvironment in response to a specific external source such as laser irradiation [18].

Near-infrared (NIR) responsive nanocarriers are extremely promising for clinical applications [19]. NIR light-mediated photothermal therapy (PTT) has been proposed as an alternative modality for hyperthermia treatment of cancers in a minimally invasive manner [20]. Indeed, cancerous cells are more sensitive to temperature increase than normal tissue cells and several tumours have a reduced tolerance to exposure temperatures in the region of 42–47°C. Hyperthermal therapy is based on this evidence and involves tumor heating within this temperature range, inducing almost irreversible damage to cells and tissues [21]. PTT relies on photothermal conversion (PTC) nano-agents [22,23] and has been developed to address the challenge related to the risk of damage to the normal tissues due to non-targeted heating in the local region [24]. Among different PTC agents [25–28], polydopamine (PDA) has been widely explored as it has shown strong NIR absorption, high PTC efficiency, excellent biocompatibility and biodegradability, and did not induce long-term toxicity [29–31]. The thermal properties of PDA make this synthetic melanin-like polymer a successful candidate for coating nanoparticles destined to combine the cancer chemo-photodynamic with photothermal therapy [32,33]. PDA has been synthesized through oxidative polymerization of dopamine to form NPs and nanocapsules. With the excellent adhesion property of PDA, the surface of polymeric nanocarrieres and inorganic NPs can be coated with a PDA shell [34]. It is possible to modify PDA surface properties so as to provide a stealth effect using polyethylene glycol (PEG) to reduce interactions with the immune system [35].

Laser-induced hyperthermal therapy combined with chemotherapy may obtain better treatment outcomes in cancer therapy [36]. The PLGA nanocarrier is highly attractive as an efficient platform for combined complementary therapeutic procedures. For example, doxorubicin-loaded PLGA/PDA core/shell NPs decorated with anti-EGFR antibody (Cetuximab) as a targeting ligand, show relevant features for photothermal therapy and chemotherapy for head and neck cancer [37].

The aim of this study was to investigate whether NIR laser-induced photothermal response can also accelerate the release of theranostic agents based on inorganic nanoparticles, i.e. CuO-NPs, from PLGA nanospheres coated with the efficient light-absorbing PDA, yielding a synergetic hyperthermia-chemotherapy combined therapeutic approach. Molecular weight (MW) of PLGA is an important formulation property that could influence degradation and release characteristics. An increasing molecular weight is leading to slower degradation rates of PLGA. Moreover, PLGA has glassy behavior with a glass-transition temperature (Tg) above 37°C, thus decrease of molecular weight of PLGA matrix results with a decrease of Tg value [38], making PLGA core more sensitive to heat generated by interaction of NIR light with PDA shell.

To address this challenge, two PLGA/PDA/PEG-based core/shell formulations from Resomer® RG 504 H (MW 38–54 kDa) and RG 502 H (MW 7–17 kDa), referred to as high MW PLGA (H-PLGA) and low MW PLGA (L-PLGA) respectively, were prepared and characterized. The photothermal effects by NIR laser irradiation were examined as a function of three different wavelengths and time. Accordingly, the laser-responsive CuO-NPs release behavior is studied by considering the PLGA molecular weight as well as its anti-cancer effect on Cal-33 head and neck cancer cells *invitro*.

## 2. Materials and methods

### 2.1. Materials

Copper acetate, dopamine hydrochloride, poly(D,L-lactide-co-glycolide) acid terminated (lactic acid (LA): glycolic acid (GA) molar ratio of 50:50, Resomer® RG 504 H (molecular weight (MW) 38–54 kDa, transition temperature (Tg) 46–50°C), and RG 502 H (MW 7–17kDa, Tg 42–46°C), referred to as high MW PLGA (H-PLGA) and low MW PLGA (L-PLGA), respectively, poly-vinyl alcohol (PVA, MW 30–70 kDa), poly(ethylene glycol) methyl ether thiol (PEG-SH, average Mn 6,000), polyethylenimine (PEI, MW 25 kDa, branched), HPLC-grade dichloromethane (DCM), dimethyl sulfoxide (DMSO), and methylthiazolyldiphenyl-tetrazolium bromide (MTT) were all purchased from Sigma-Aldrich Chemicals, Israel. Copper (II) sulfate pentahydrate and glacial acetic acid were obtained from Carlo Erba, Spain. Sodium hydroxide was purchased from Bio-Lab Ltd, Israel. Tris base (2-amino-2-(hydroxymethyl)-1,3-propanediol was obtained from Amresco, USA. 0.9% sodium chloride intravenous solution for infusion (saline) was obtained from B. Braun. Cell culture media and supplements were purchased from Biological Industries, Israel. LC/MS-grade water was used throughout the encapsulation process. For all other purposes, Milli-Q water with a resistivity of 18.2 MΩ cm was used.

### 2.2. Preparation of PEGylated polydopamine-coated PLGA nanospheres encapsulated CuO-NPs

*Synthesis of CuO-NPs loaded PLGA nanospheres*: CuO-NPs with an average size of ~7 nm were synthesized by a simple solution technique as previously described [8]. Then, CuO-NPs were encapsulated in nanospheres made of PLGA polymer (Resomer® RG 504 H or RG 502 H) using the double emulsion (W_1_/O/W_2_) and subsequent solvent evaporation method, based on our previous study [15]. Briefly, 1 mL of aqueous dispersion as-synthesized of CuO-NPs (3.70 mg Cu/mL) was added to the organic phase composed of 100 mg PLGA dissolved in 10 mL dichloromethane. The mixture underwent ultra-sonication using a probe-type sonicator (3 min., 20–50 W, Q700, Qsonica, USA) in an ice-water bath. The formed W_1_/O emulsion was immediately mixed with 10 mL of 1% (w/v) poly-vinyl alcohol (PVA, MW 30–70 kDa) aqueous solution and emulsified by sonication under the same conditions to form the double (W_1_/O/W_2_) emulsion. Then, the emulsion obtained was poured into 180 mL of 0.1% (w/v) PVA aqueous solution and stirred for 3 hours at room temperature to evaporate the DCM. The nanospheres were collected by centrifugation (12,000 rpm, 20 min.) and washed twice with deionized water. The aqueous dispersion nanospheres (4 mL) were stored at 4°C and used for the next step in less than 1 day.

*Surface modification by PEGylated polydopamine*: The coating procedure was adopted with modifications [39]. First, 4 mL suspension of CuO-NPs@PLGA NS was added to 48 mL Tris buffer (10 mM, pH 8.5), and then mixed with 24 mg dopamine hydrochloride. The mixture was left stirring in an open beaker at room temperature for 3 hours. PDA-coated nanospheres were obtained by centrifugation (12,000 rpm, 15 min.) and water washing. Second, the resulted suspension of CuO-NPs@PLGA/PDA NS (4 mL) was added to 50 mg PEG-SH previously dissolved in 4.8 mL Tris buffer (10 mM, pH 8.5). The mixture was stirred by orbital shaking (160 rpm) overnight, then centrifuged (12,000 rpm, 15 min.) and washed twice with water. Finally, the surface-modified nanospheres (CuO-NPs@PLGA/PDA/PEG) were dispersed with 4 mL water and stored at 4°C.

### 2.3. Determination of CuO-NP content

Inductively coupled plasma-mass spectroscopy (ICP-MS) analysis was used to determine the amount of CuO in the starting material (CuO-NPs) and that of CuO encapsulated in the PLGA/PDA/PEG NS. The weight of loaded CuO-NPs@PLGA/PDA/PEG NS was determined after freeze drying. The encapsulation efficiency (EE%) and loading capacity (LC%) were calculated using the following equations:

(1) $$EE\left(\%\right)=\frac{weight\;of\;encapsulated\;CuO}{weight\;of\;CuO\;initially\;loaded}\;\times 100$$

(2) $$LC\left(\%\right)=\frac{weight\;of\;encapsulated\;CuO}{weight\;of\;CuO@PLGA/PDA/PEG}\;\times 100$$

### 2.4. Characterization

The size and shape of the nanospheres were analyzed by cryogenic transmission electron microscopy (cryo-TEM). Vitrified samples were examined in a FEI T12 G2 cryo-TEM (Netherlands) operating at 120 kV, using a Gatan 626 cryo-holder (USA). Images were recorded in a Gatan US1000 high-resolution cooled CCD camera (USA) and were processed with DigitalMicrograph version 3.3.1 software. The ramp-shaped optical density gradients in the background were digitally corrected. Particle size distribution (mean hydrodynamic, diameter, and polydispersity index) and zeta potential were determined by the ZetaSizer (ZetaSizer Nano ZS, Malvern Panalytical, UK). For each sample, five measurements were performed without a time delay. Total content of copper (Cu) in the samples was determined with ICP-MS (7900 ICP-MS Agilent Technologies, USA). Thermal behavior of the nanospheres was examined using a Q500 Thermogravimetric Analyzer (TA Instruments, USA). Samples were placed in a platinum crucible and heated from 35°C to 600°C at a heating rate of 10°C/min under nitrogen atmosphere. X-ray photoelectron spectroscopy (XPS) spectra were measured using Kratos AXIS Supra spectrometer (Kratos Analytical Ltd., UK) with an Al Kα monochromatic radiation X-ray source (1486.7 eV). Data were collected and analyzed using the ESCApe processing program (Kratos Analytical Ltd.) and Casa XPS (Casa Software Ltd.). The XPS spectra were acquired with a takeoff angle of 90⁰ (normal to analyzer), the vacuum condition in the chamber was 2 × 10^−9^ Torr, and the survey spectra were measured with pass energy 160 eV and 1 eV step size, and high-resolution XPS spectra with pass energy of 20 and 0.1 eV step size. The binding energies were calibrated using C 1s peak energy as 285.0 eV. Ultraviolet–visible (UV–vis) spectroscopy (Ultraspec 2100 pro-UV-visible spectrophotometer, Amersham Biosciences, USA).

### 2.5. Assessment of the photothermal effect

The photothermal effect of CuO-NPs@L-PLGA/PDA/PEG and CuO-NPs@L-PLGA was evaluated after irradiation with laser sources within the NIR range. The samples were diluted in distilled water with a concentration of 0.033 mg CuO/mL and placed in 1.5 mL Eppendorf tubes (see section 3.2.). For control measurements, distilled water samples were used. Diode lasers with wavelengths of 808 nm, 940 nm, and 1064 nm were employed to irradiate the aqueous solutions of CuO-NPs@PLGA and CuO-NPs@PLGA/PDA/PEG nanospheres in a continuous wave mode. Laser light was guided inside the samples through a 300 μm-diameter quartz optical fiber. Each experiment was repeated three times at the same laser settings; i.e. laser power of 2.5 W, and irradiation time of 120 s. Temperature measurements were performed during the laser irradiation procedure with optical fiber sensor-based thermometers (Fiber Bragg Gratings, FBGs) [40]. Two optical fibers were immersed in the sample, parallel to each other at a 2 mm distance from the laser-guiding fiber (see section 3.2.). The FBGs were inscribed in single-mode optical fibers using the femtosecond point-by-point writing technology to produce highly dense FBG arrays [41]. Each fiber embeds an array of 25 FBGs that act as sensing points and have the following properties: a 0.9 mm grating length and a 0.1 mm edge-to-edge distance between gratings. As a result, the sensors have a 1.0 mm spatial resolution along a 25 mm sensing length. The temperature sensitivity of the sensors was obtained with calibration in a thermal chamber in a temperature range from 30°C to 140°C. The reflection spectrum of the FBGs was measured by the Micron Optics si255 interrogation unit (Micron Optics, Atlanta, USA) and analyzed to obtain a temperature profile along the optical fiber.

### 2.6. Determination of heating efficiency

The heating efficiency (HE) of the CuO-NPs@H-PLGA/PDA/PEG (coated NS) versus control (water) was assessed, and expressed as the ratio of the temperature difference between CuO-NPs@H-PLGA/PDA/PEG (uncoated NS) and control, and the maximum temperature increase measured by the control (Eq. 3). Similarly, HE of the CuO-NPs@H-PLGA (uncoated NS) versus control was assessed and expressed according to Eq. 4. The photothermal properties of PDA were also assessed by calculating HE of the CuO-NPs@PLGA/PDA/PEG (coated NS) over the CuO-NPs@PLGA (uncoated NS), according to Eq. 5.

(3) $$HE_{coated\;NS/water}\left(\%\right)=\frac{T_{CuO@PLGA/PDA/PEG}\;-\;T_{water}}{T_{water}}\;\times \;100$$

(4) $$HE_{uncoated\;NS/water}\left(\%\right)=\frac{T_{CuO@PLGA}\;-\;T_{water}}{T_{water}}\;\times 100$$

(5) $$\;HE_{coated\;NS/uncoated\;NS}\left(\%\right)=\frac{T_{CuO@PLGA/PDA}\;-\;T_{CuO@PLGA}}{T_{CuO@PLGA}}\;\times 100$$

### 2.7. In vitro evaluation of laser-response copper release

As stated above, the highest photothermally induced temperature elevation of aqueous suspension of CuO-NPs@H-PLGA/PDA/PEG NS was obtained under laser irradiation at wavelength of 808 nm. Thus, this wavelength was employed to study the release of CuO-NPs from the coated nanospheres. Samples (1.5 mL of saline) of CuO-NPs@H-PLGA/PDA/PEG or CuO-NPs@L-PLGA/PDA/PEG formulations (~50 µg Cu/mL) were placed in 24-well plates. The focal spot of the laser beam was set at the center of the suspension (circular diameter of ~1 cm, distance of ~1 cm). At appropriate intervals, a NIR light (i.e. 808 nm wavelength) was delivered through an optical fiber with laser power of 2.5 W. The samples were exposed to six cycles of laser irradiation of 30 s each. Irradiation time points were at 0, 45, 67, 112, 197 and 217 s for CuO-NPs@H-PLGA/PDA/PEG and 0, 30, 47, 91, 126, 144 s for CuO-NPs@L-PLGA/PDA/PEG). After cycle nos. 1, 3, 4, and 6, the suspension was transferred to a plastic tube and centrifuged at 12,000 rpm for 3 min (at 22°C). Then, 1 mL of the supernatant was removed and immediately replaced with fresh medium to keep a constant volume for the next irradiation cycle. Finally, the samples were incubated at 37°C under orbital shaking (260 rpm) and the released copper was determined after 3 days. The released CuO-NPs concentration (in the form of copper) in the supernatant was determined by polyethylenimine (PEI) colorimetric method [16]. The supernatant was mixed vigorously with 0.110 mL PEI solution (188 mg/mL). Then the absorbance was measured at λ_max_ = 275 nm and Cu content was calculated against a standard calibration curve of UV-vis detection [16]. Data obtained in triplicate were analyzed and percent copper release versus time was plotted. To evaluate the temperature increasing during the laser irradiation of the nanosphere formulation, infrared thermal camera videos recorded the changes in the temperature of the formulation suspension during the whole irradiation (at a rate of 6 frame per second). From the recorded video, the average temperature over time was extracted by defining a region of interest (ROI) at the center of each well.

Control experiments were performed using a similar procedure, but without laser irradiation. Specifically, samples in plastic tubes were incubated at 37°C under orbital shaking (140 rpm) and the amount of released copper was determined at each predetermined time point (e.g. 48 hours) as described above.

The CuO-NPs release rate and mechanism from coated nanospheres were analyzed using following mathematical models: zero-order release kinetics, first-order release kinetics, Higuchi model and Korsmeyer-Peppas model *(K_0_, K_1_, K_H_*, and *K_P_* are model release constants, respectively), as described previously [42]. The calculated values of regression coefficient (R^2^) were used to correlate with the best model to describe the copper release behavior. The release exponent *(n*), which was estimated from Korsmeyer-Peppas equation, allowing determination of the diffusional parameters for spherical encapsulation shape: Fickian diffusion (n ≤ 0.43), non-Fickian or anomalous transport (0.43 < n < 0.85), and mainly erosion release controlled (n ≥ 0.85) [43].

### 2.8. Cell viability assay

Cell viability was performed through standard MTT assay. 5 × 10^4^ Cal-33 head and neck squamous cell carcinoma cells were seeded in two separate 96 well plate. 24 h after seeding, the cells were incubated (5% CO_2_ at 37°C) with CuO-NPs@H-PLGA/PDA/PEG or CuO-NPs@L-PLGA/PDA/PEG formulations at three concentrations (110, 55 and 27.5 μg Cu/mL). Then, the cells in each experimental well of one plate, were exposed to 808 nm laser irradiation (2.2 W) for 40 s, while the other plate was not irradiated. After 48 h the medium was removed and 5% MTT solution in growth medium was added. After additional 1.5 h the solution was removed and DMSO was added. Cell viability was evaluated by measuring the absorbance of each well at 570 nm relative to control wells with BioTek H1M plate reader. Experiments were done in triplicates. Unpaired *t-test* was performed with GraphPad Prism-9 to measure statistical significance.

### 2.9. In vitro MR imaging measurements

MRI scans were conducted upon identical containers with CuO concentrations ranging from 0 to 280 µg/mL. The scans were performed using a 9.4 T preclinical MRI system (Bruker, Germany). The implemented protocol was the rapid acquisition relaxation enhanced (RARE) T1-weighted sequence. The following scanning parameters were used: Echo time (TE) = 50 ms, Repetition times (TR) = 750, 1000, 1200, 1500, 3000, 4000 ms, field of view (FOV) = 4.8 cm × 4.8 cm, matrix 160 × 160 pixel, slice thickness = 2.5 mm, and average number = 1. Following acquisition, images mapping the T1 values per pixel were reconstructed using an exponential curve fitting for each pixel, using MATLAB®, Bruker Paravision 5 software. The contrast-enhancing effect was next quantified by selecting a ROI in the T1 mapping image and subtracting the mean T1 value obtained at a lower concentration from the averaged T1 value at the higher concentration and dividing the result by the T1 value obtained for the lower concentration.

## 3. Results and discussion

### 3.1. Characterization of PEGylated polydopamine-coated PLGA nanospheres encapsulated CuO-NPs

The CuO-NPs loaded PLGA NS were prepared as a scaffold, tailored by coating shells of PDA and PEG. To evaluate the PLGA potential to act as a component of a photothermal controlled delivery system for CuO-NPs, two different molecular weights (MW) were selected: Resomer® RG 504 H (MW 38–54 kDa) and RG 502 H (MW 7–17kDa), referred to in this study as high MW PLGA (H-PLGA) and low MW PLGA (L-PLGA), respectively. Obviously, apart from the MW, it is of the utmost importance to keep all other polymer properties and process parameters unchanged. The encapsulation process of CuO-NPs was obtained by using a double emulsion-evaporation procedure (water/oil/water emulsion) [44]. Then, the surface of the nanospheres was coated through oxidative polymerization of dopamine under alkaline conditions of a Tris buffer to form a thin primer layer of PDA. A second coating was achieved by binding poly(ethylene glycol) methyl ether thiol (PEG-SH) to PDA through the Michael addition reaction [45]. Subsequently, two formulation types of multifunctional nanospheres were obtained, containing higher MW (H-PLGA) and lower MW (L-PLGA) building blocks and referred to as CuO-NPs@H-PLGA/PDA/PEG and CuO-NPs@L-PLGA/PDA/PEG, respectively. CuO-NPs encapsulation efficiency and CuO-NPs loading capacity values of both multifunctional nanosphere formulations are summarized in Table 1. The L-PLGA-based formulation compared to the H-PLGA-based formulation exhibited higher encapsulation efficiency and similar loading capacity. This can be attributed to low MW PLGA causing a decrease of organic phase viscosity during the encapsulation process [46].

**Table 1.** Encapsulation efficiency (EE) and loading capacity (LC) of the coated-nanosphere formulations

Formulation	EE^(a)^ (%)	LC^(a)^ (%)
CuO-NPs@H-PLGA/PDA/PEG	24.6 ± 1.5	2.7 ± 0.5
CuO-NPs@L-PLGA/PDA/PEG	34.5 ± 3.2	3.6 ± 0.1

Results represent the mean standard deviation of representative batches (n = 3);

^(a)^The values were calculated based on copper content quantified by the ICP analysis

**Figure 1.**
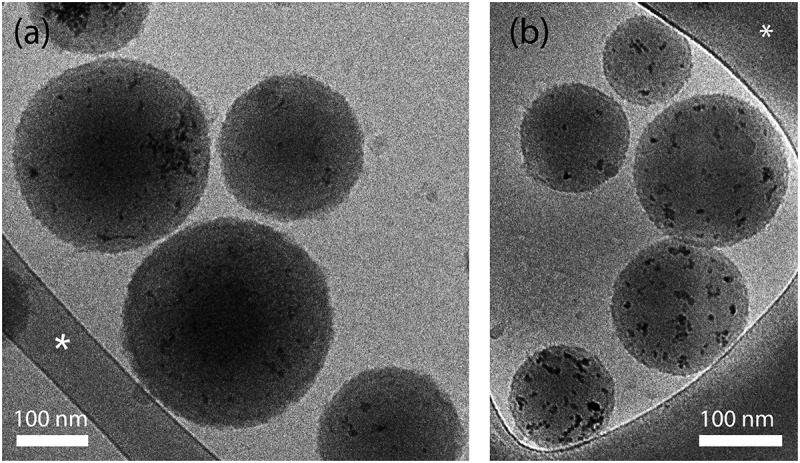
Cryogenic-TEM images of coated nanospheres. (a) CuO-NPs@H-PLGA/PDA/PEG and (b) CuO-NPs@L-PLGA/PDA/PEG in water. Scale bar denotes 100 nm. The asterisk denotes perforated carbon film supported on a TEM copper grid

Nanospheres with spherical morphology were observed by cryo-TEM. As shown in Figure 1a,b, the CuO-NPs were successfully encapsulated in the H-PLGA and L-PLGA matrices. It can also be noticed that the surface of nanospheres are coated with a very thin rough film compared to uncoated CuO@PLGA NS, which are characterized by a smooth surface as we had shown in a previous study [16]. This thin film (approx. 2 nm as measured by TEM), attributed to the formation of PDA/PEG shells, is also consistent with another work studying PLGA coating with similar dopamine concentration (0.5 mg/mL) [39]. The cryo-TEM images revealed that most nanospheres had a diameter 150–250 nm for both formulations, which appeared to be smaller than the average hydrodynamic diameter (z-average) measured by DLS. Since these particle size values are based on a light scattering method, the results may be skewed toward larger particles [47,48]. The average hydrodynamic diameter and the polydispersity index (PDI) of CuO-NPs@H-PLGA/PDA/PEG NS were 288 nm and 0.201, respectively, while for the CuO-NPs@L-PLGA/PDA/PEG NS they were found to be 257 nm and 0.143, respectively (Table 2). The size and size distribution (PDI) of coated nanospheres increased, compared to those of uncoated nanospheres, due to the higher hydrophilic nature of PDA and PEG. CuO-NPs were characterized by a higher positive net charge (> 35 mV) due to the acetate ligands on the surface [49], resulting in a high aqueous dispersibility. Following their encapsulation in PLGA, zeta potential values dropped to −18.7 mV and −22.2 mV for CuO-NPs@H-PLGA NS and CuO-NPs@L-PLGA/PDA/PEG NS, respectively (Table 2). After coating, a slight decrease of net charges was observed. These negative values can be attributed to the carboxylate moiety on the polymer, whereas the negative value of the coated nanospheres may arise from the deprotonation of the catechol groups of the PDA in water. This provides the electrostatic stabilization needed for further application. Accordingly, the obtained multifunctional nanocarriers CuO-NPs@H-PLGA/PDA/PEG and CuO-NPs@L-PLGA/PDA/PEG address the most important properties for passive targeting through EPR effects; i.e. particle size should be between 10 and 400 nm, and a neutral or negative surface charge should reduce size effects and toxicity and possess a shielding element against opsonization and phagocytosis such as can be achieved by PEGylation [50].

**Table 2.** Properties of the multifunctional nanosphere formulations

Formulation	Particle Size^(a)^ (nm)	PDI^(a,b)^	Zeta Potential (mV)
CuO-NPs@H-PLGA	251 ± 28	0.115 ± 0.063	−18.7 ± 1.0
CuO-NPs@H-PLGA/PDA/PEG	288 ± 11	0.201 ± 0.047	−15.4 ± 1.5
CuO-NPs@L-PLGA	240 ± 16	0.106 ± 0.028	−22.2 ± 1.3
CuO-NPs@L-PLGA/PDA/PEG	257 ± 23	0.143 ± 0.014	−22.7 ± 1.4

Results represent the mean and standard deviation of representative batches (n = 3);

^(a)^Data was recorded by dynamic light scattering (DLS) as Z-average hydrodynamic diameter; ^(b)^ Polydispersity index

**Figure 2.**
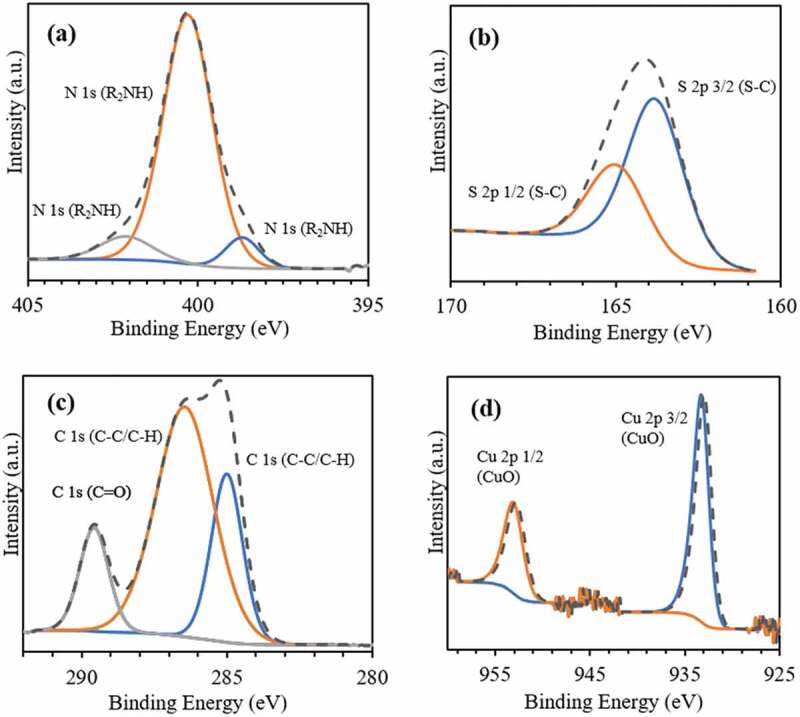
XPS spectra of the coated nanospheres (CuO-NPs@H-PLGA/PDA/PEG NS). (a) Spectrum in the N region, (b) Spectrum in the S region, (c) Spectrum in the C region and (d) Spectrum in the Cu region

X-ray photoelectron spectroscopy (XPS) analysis was used to provide an additional indication of the surface enrichment, verifying the formation of PDA/PEG coating. XPS reveals that for both nanosphere formulations, the elements C, O, N, S, and Cu have similar atomic concentrations in the near-surface region (Table 3). Figure 2 displays the XPS spectra of CuO-NPs@H-PLGA/PDA/PEG NS. The peaks obtained at binding energies of 398.7, 400.3, and 402.1 eV (Figure 2a) can be assigned to the = NR, R2NH, and R-NH2 groups, respectively [51]. These nitrogen-containing functional groups are attributed to the PDA coating shell. The binding of PEG chains through thiol groups is evident from S 2p spectrum (Figure 2b). The peaks obtained at binding energies of 163.8 and 164.98 eV are assigned to S 2p(3/2) and S 2p(1/2), respectively, and are related to the covalently bonded sulfur (C–S) species [52]. In the C 1s region (Figure 2c), peaks at binding energies of 285, 286.4, and 289.6 eV are assigned to the (C-C), C-O/C-N, and C = O species, respectively. As for XPS analysis, the generally accepted information depth is 10 nm, while low contents of Cu^2+^ are also observed. Figure 2d exhibits the peaks at binding energies of Cu 2p(3/2) and Cu 2p(1/2) at 933.3 and 953.1 eV, respectively, attributed to the presence of CuO-NPs [53]. The spectra of CuO-NPs@L-PLGA/PDA/PEG are similar and not shown for brevity.

**Table 3.** Atomic concentrations by XPS analysis

Formulation	Atomic concentration [%]				
	C	O	N	S	Cu
CuO-NPs@H-PLGA/PDA/PEG	64.6	32.5	2.60	0.05	0.16
CuO-NPs@L-PLGA/PDA/PEG	63.8	33.9	2.12	0.03	0.07

**Figure 3.**
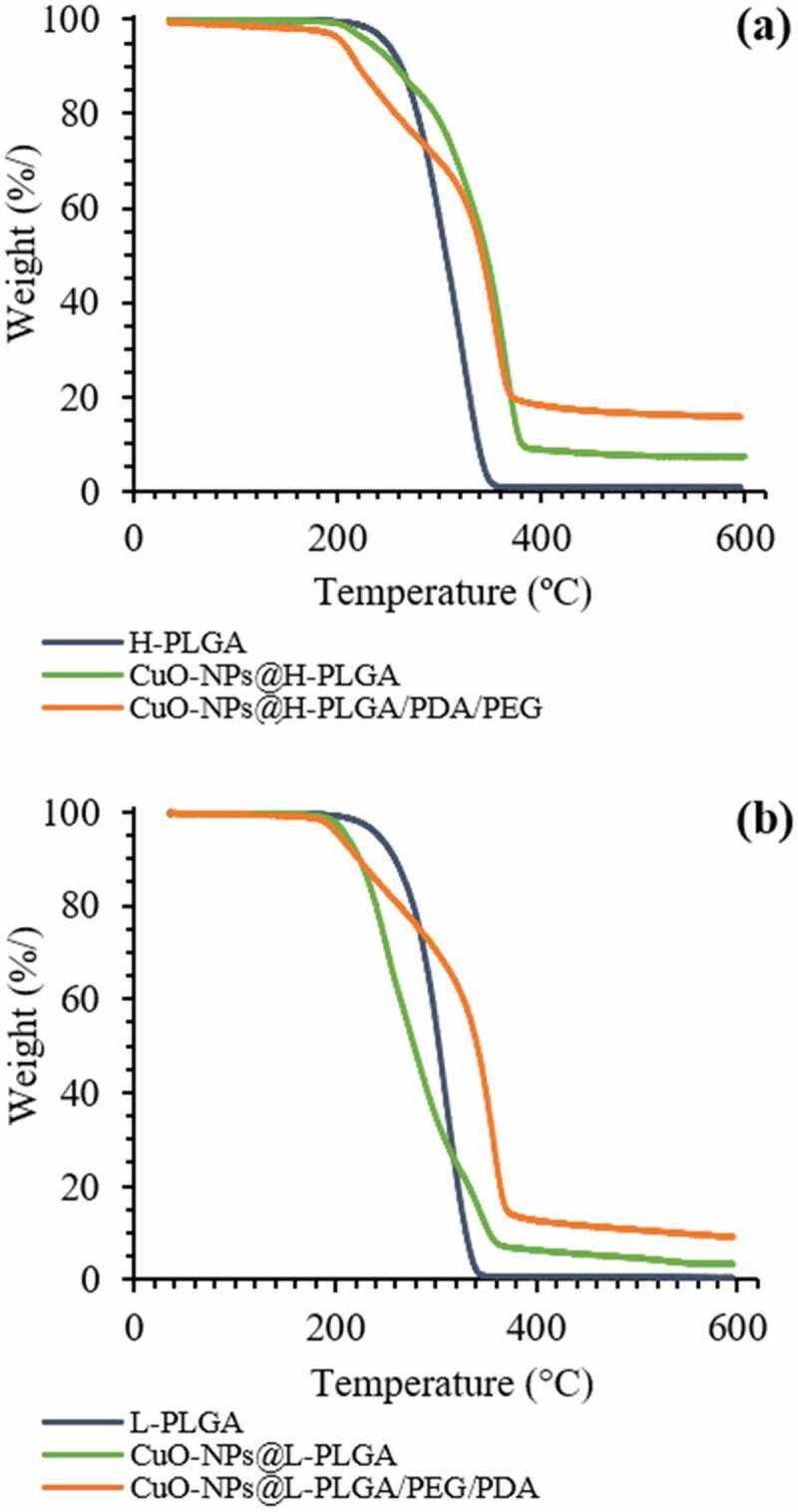
TGA of nanospheres with and without coating compared to the neat polymer. Nanospheres were formed with (a) H-PLGA and (b) L-PLGA

To examine the thermal stability of the nanosphere formulations, thermal gravimetric analysis (TGA) was conducted. Figure 3 shows TGA curves over the temperature range of 35–600°C for neat PLGA polymers (starting materials) and CuO-NPs loaded PLGA NS, before and after coating. Both neat PLGA polymers show a single weight-loss stage attributed to thermal decomposition. The H-PLGA and L-PLGA degraded in the temperature range of ~190 – 350°C. In the presence of encapsulated CuO-NPs, the onset temperatures of decomposition for both H-PLGA NS and L-PLGA NS did not change significantly. However, the loaded NS show more complex thermal decomposition behavior when compared to the neat PLGA, similar to that observed in our previous work [15]. These results show that both formulations of coated NS are thermally stable up to almost 190°C, and are suitable for laser irradiation procedures. Mass balance shows approximately 7% and 5% residual weight, relative to the weight of the encapsulated CuO-NPs in nanospheres made by H-PLGA and L-PLGA, respectively. After decomposition of CuO-NPs@H-PLGA/PDA/PEG and CuO-NPs@L-PLGA/PDA/PEG, the mass residues were close to 16% and 9%, respectively. The higher mass residues are mostly related to char formation from decomposition of PDA under inert conditions. Therefore, CuO loading capacities of coated nanosphere formulations were determined by ICP-MS analysis.

### 3.2. Evaluation of photothermal effects of the polydopamine coating

**Figure 4.**
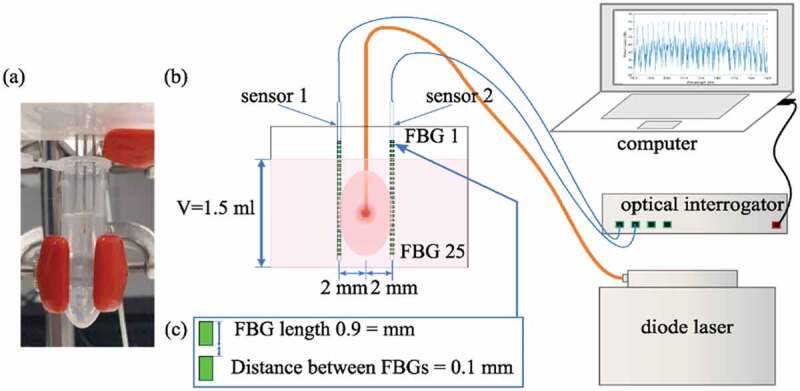
Schematic overview of the experimental setup. (a) Digital photo showing the sample holding setup, (b) Temperatures measured by the FBG sensors at a 2 mm distance from the laser applicator and (c) Zoom-in of the schematic illustrating spatial characteristics of the sensors

**Figure 5.**
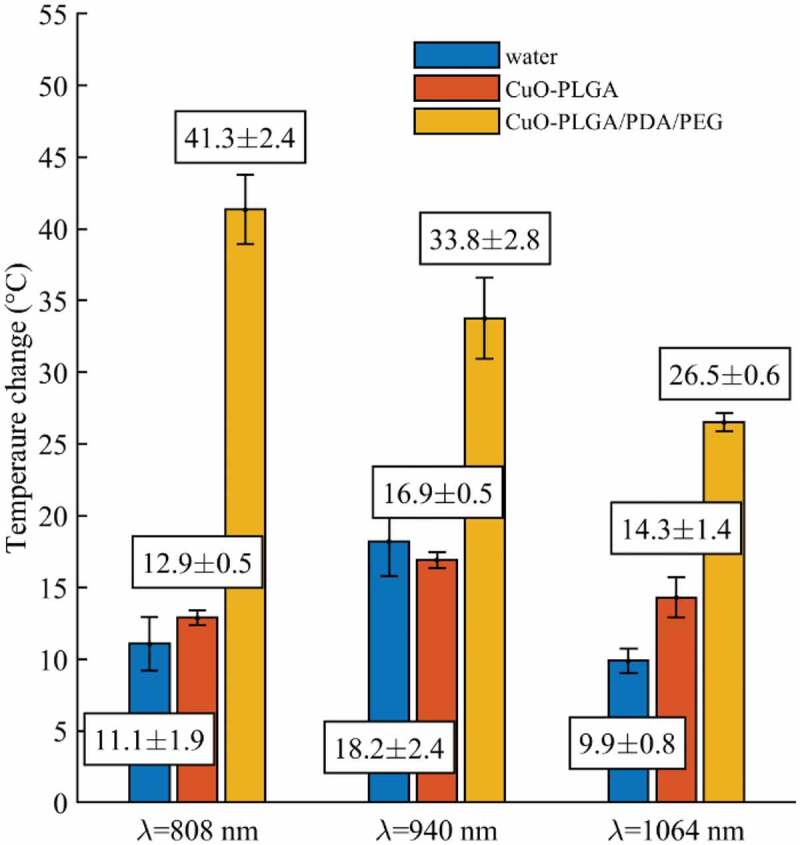
Peak temperature elevations measured during laser irradiation (λ = 808 nm, 940 nm, and 1064 nm) of water (control), CuO-NPs@H-PLGA, and CuO-NPs@H-PLGA/PDA/PEG

The photothermal effects of the coated NS encapsulated in CuO-NPs were evaluated by means of FBG sensors, which were able to measure the temperature profile generated by the laser interaction with the samples (Figure 4 and section 2.5). Figure 5 shows the maximum measured temperature induced by the laser irradiation of the CuO@H-PLGA/PDA/PEG and CuO@PLGA nanospheres-based samples and water (as a control) for three different wavelengths studied (i.e. 808 nm, 940 nm, and 1064 nm). These results demonstrated the significant effect of the PDA shell in heat induction when the target was irradiated with the NIR-laser beam. Moreover, among the three mentioned wavelengths, the CuO@H-PLGA/PDA/PEG NS exhibited an excellent photothermal heating efficiency under NIR laser irradiation at 808 nm. Conversely, the composite of CuO@PLGA did not show any significant increase in temperature in comparison to the water. Thus, the uncoated NS could not transform light to heat and cannot be used as a photothermal agent. The results show that for water (control), the mean temperature elevation values were 11.1°C, 18.2°C, and 9.9°C, at 808 nm, 940 nm, and 1064 nm, respectively. The highest temperature elevation for water was experienced at 940 nm, and the peaks in temperature for the other two wavelengths were similar. These results are in agreement with the water absorption coefficients for different wavelengths [54].

The photothermal effects of the CuO-NPs were evaluated using the indices for the heating efficiency (HE), which were defined in Eqs. 3, 4, and 5. At first, the HE of both CuO@H-PLGA/PDA/PEG and CuO@H-PLGA versus water control is reported (Eqs. 3 and 4). NIR-laser irradiating of the sample of CuO@H-PLGA/PDA/PEG NS induced a temperature increase of 30.2°C (11.1°C to 41.3°C, corresponding to HE = 272%), 15.6°C (18.2°C to 33.8°C, corresponding to HE = 86%), and 16.6°C (9.9°C to 26.5°C, corresponding to HE = 168%) for the wavelengths of 808 nm, 940 nm, and 1064 nm, respectively. For this sample, the maximum obtained temperature corresponds to the wavelength of 808 nm; i.e. 41.3 ± 2.4°C. The measured temperature and the HE values prove that CuO@H-PLGA/PDA/PEG NS have a significant thermal effect over water which served as control.

Compared to water, CuO@H-PLGA exhibited a temeperature elevation of 1.8°C (11.1°C to 12.9°C, HE = 16%), and 4.4°C (9.9°C to 14.3°C, corresponding to HE = 44%) for the wavelengths of 808 nm and 1064 nm, respectively. In the case of 940 nm, the temperature exhibited by the CuO@H-PLGA sample; i.e. 16.9°C, was also similar to the values measured in water; i.e. 18.2°C. The measured temperature and the resulting HE values demonstrate that CuO@H-PLGA NS behaves almost the same as water from the point of view of photothermal effect; hence, no significant thermal effect was measured. Contrary to that, the thermal effect achieved with the photothermal properties of PDA was much more substantial and is also shown in terms of HE, by comparing the maximum temperature measured in CuO@H-PLGA/PDA/PEG *vs*. CuO@H-PLGA, during laser irradiation (Eq. 5). In this case, we measured an increase of 28.4°C (12.9°C to 41.3°C, corresponding to HE = 220%), 16.9°C (16.9°C to 33.8°C, corresponding to HE = 100%), and 12.2°C (14.3°C to 26.5°C, corresponding to HE = 85%) for the wavelengths of 808 nm, 940 nm, and 1064 nm, respectively. These data demonstrate the photothermal effect of CuO@H-PLGA/PDA/PEG NSs over the uncoated particles. Table 4 summarizes the results of the HE for the nanosphere formulations.

**Table 4.** Comparative evaluation of the heating efficiency (HE) for light absorbance by the nanosphere formulations

Wavelength (nm)	HE _coated NS/water_ ^(a)^ (%)	HE _uncoated NS/water_ ^(a)^ (%)	HE _coated NS/uncoated NS_ ^(b)^ (%)
808	272	16	220
940	86	~0	100
1064	168	44	85

Results are calculated with respect to the mean maximum temperature shown in Figure 5.

^(a)^HE of the CuO-NPs@H-PLGA/PDA/PEG and CuO-NPs@H-PLGA *vs*. water was calculated using Eq. 3 and 4; ^(b)^ HE of the CuO-NPs@H-PLGA/PDA/PEG *vs*. CuO-NPs@H-PLGA, calculated using Eq. 5.

### 3.3. In vitro evaluation of photothermal controlled copper release

#### 3.3.1. Temperature evaluation

**Figure 6.**
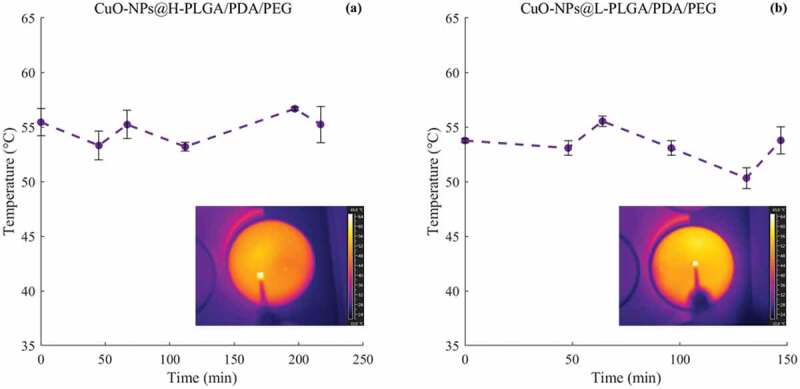
Temperature measurements of coated nanospheres in response to 808 nm laser irradiation over time. (a) Temperature experienced by CuO-NPs@H-PLGA/PDA/PEG samples in saline media, (b) Temperature experienced by CuO-NPs@L-PLGA/PDA/PEG samples in saline media. The corresponding thermal maps obtained for the last frame for the two cases are also depicted

During the laser-controlled copper release experiments, the temperature reached in the samples was monitored. Results show that the temperature experienced by the samples ranges from 50 to 60°C for both coated-nanosphere formulations. In other words, repeated laser irradiations do not alter the photothermal response of the CuO-NPs, which is maintained within the defined temperature range (Figure 6). Hence, regardless of their molecular weight, the temperature raised by the solutions is compatible with the typical range suitable for photothermal therapy. Moreover, the temperature is influenced mainly by the PDA coating.

#### 3.3.2. Copper release behavior

The pH-dependent drug release profiles from PDA-coated nanocarriers or from PDA spheres have been extensively investigated [34]. In this study the *in vitro* release experiments were conducted in a saline medium only, at pH ~5.5 which has favorable environment for antitumor efficiency. In these acidic conditions (pH <6), the copper complex with the catechol (*ortho* bi-phenol) groups of PDA does not form [55,56]; hence, an accelerated CuO-NPs release is expected.

**Figure 7.**
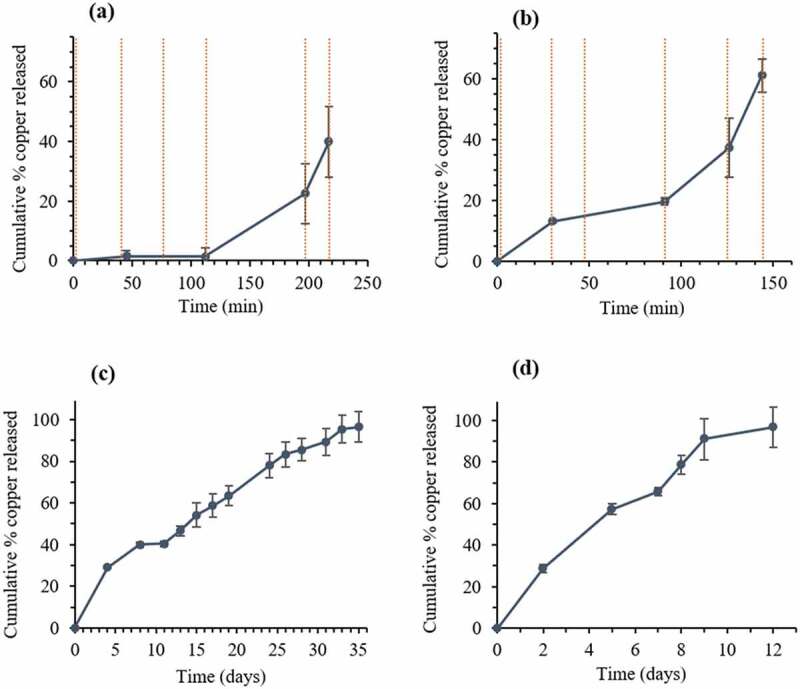
*In vitro* release profiles of copper from coated nanospheres in saline. CuO-NPs@H-PLGA/PDA/PEG (a and c) and CuO-NPs@L-PLGA/PDA/PEG (b and d). With laser irradiation (a and b) and at 37°C without laser irradiation (c and d). The stimulated release using six cycles of laser pulse (808 nm, 2.5 W, 30 s) indicated by vertical lines. Each point represents the mean ± SD (n = 3). Note that the release timescale of the non-irradiated samples is in days as opposed to minutes for the irradiated samples

Cumulative amounts of CuO-NPs released from the coated nanospheres have increased with lengthier exposure time to laser irradiation at constant power (Figure 7a, b), and with an increase of incubation time at 37°C without laser irradiation (Figure 7c, d). After applying a power of 2.5 W for 30 s in six cycles, almost 40% of the loaded copper was released from the H-PLGA/PDA/PEG NS samples during 217 min., compared to 128 min. observed for L-PLGA/PDA/PEG NS samples. In the absence of laser treatment, a release of approximately 40% of copper was observed only after 8 days and 5 days of incubation at 37°C for H-PLGA/PDA/PEG NS and L-PLGA/PDA/PEG NS, respectively. It is clearly observed that the laser stimulates the copper release, and that without laser irradiation, the copper is released to the medium at a much slower rate. The light-induced response can be attributed to the PDA coating as photothermal sensitive polymer due to its broad absorption spectrum, especially in the first biological optical window (650–950 nm), hence allowing increase of temperature at levels useful for hyperthermia therapy [30]. Additionally, Viger et al. suggested a release mechanism from PLGA NS (980 nm, 1 W) relying on excitation of confined water. Hence, entrapped water plays a crucial role in payload release by absorbing the NIR light to induce localized heating inside the particles [57].

Coated nanospheres composed of low MW PLGA (CuO-NPs@L-PLGA/PDA/PEG) show 61% release of copper after 6 cycles of laser pulses (during 144 min.). Incubation at 37°C for an additional 3 days yields a total release of 93% of the copper indicating a sustained effect. Compared to CuO-NPs@L-PLGA/PDA/PEG NS, the CuO-NPs@H-PLGA/PDA/PEG NS responded substantially less to laser irradiation exposure (808 nm, 2.5 W for 30 s), showing only 40% release of the payload after 6 cycles of laser pulses (during 217 min.) and a much lower total percentage of copper release (48%) after three additional days of incubation (at 37°C). The results suggest that the release kinetics of copper from coated nanospheres under laser irradiation are relative to the MW of the PLGA core. The effect of polymer chain size on the release behavior can be ascribed to glass transition temperature (Tg) [58] of the PLGA RG 502 H and PLGA RG 504 H, reported to be 42–46°C and 46–50°C, respectively. Therefore, under laser irradiation, when the temperature is increased to 50–60°C (Figure 6) that is, above the Tg values accelerated diffusion of the CuO-NPs from the matrix is observed. The effect is more pronounced with low MW as its Tg is slightly lower and chain mobility is higher. According to TGA analysis (Figure 3), at this temperature range, thermal decomposition does not occur. However, it should be noted that the combination of heat and water environment can influence the degradation process of this PLGA-based delivery system [59].

As described above, the laser-responsive release behavior is mainly dependent on the PLGA core, resulting in faster release of the embedded CuO-NPs with lower MW of the polymeric core (Figure 7a,b). This can be supported by the XPS analyses (Figure 2), showing that physiochemical differences between two nanosphere types mainly arose from their PLGA core. This behavior was also found in cumulative copper release profiles without exposure to laser irradiation (Figure 7c,d). More than 96% of the loaded copper was released within 12 days from CuO-NPs@L-PLGA/PDA/PEG NS, compared to 35 days from CuO-NPs@H-PLGA/PDA/PEG NS. To provide a correlation between the experimental data and drug release kinetics models, the *in vitro* release data of coated nanospheres were analyzed using mathematical equations (section 2.7) and summarized in Table 5. The Korsmeyer-Peppas model was found to best fit the CuO-NPs@H-PLGA/PDA/PEG NS formulation (R^2^ = 0.989) and displays a good correlation for CuO-NPs@L-PLGA/PDA/PEG NS (R^2^ = 0.932). The transport mechanisms may be determined using the values of *n* in the Korsmeyer–Peppas model (as detailed in section 2.7). The *n* values found for both NS formulations were 0.85 > *n*> 0.43, suggesting that the release is mainly non-Fickian or anomalous transport, meaning that the release mechanism is governed by both erosion and diffusion. This was also confirmed by the Higuchi model describing copper release as a diffusion process for CuO-NPs@H-PLGA/PDA/PEG NS and CuO-NPs@L-PLGA/PDA/PEG (both R^2^ = 0.971). In addition, the data fit zero-order kinetics for CuO-NPs@H-PLGA/PDA/PEG NS (R^2^ = 0.968) as similarly found for uncoated CuO-NPs@PLGA NS in our previous work [16]. Compared to the uncoated NS exhibiting a monophasic profile (R^2^ > 0.99), the coated-NS release profile shows an initial rapid release stage (Figure 7b) that can be attributed to the surface modification by a hydrophilic nature PEG coating.

**Table 5.** Fitting release kinetic models to copper release data for coated nanospheres ^(a).^

Model	Zero order		First order		Higuchi		Korsmeyer–Peppas	
Formulation	R^2^	K_0_	R^2^	K_1_	R^2^	K_H_	R^2^	*n* ^(b)^
CuO-NPs@H-PLGA/PDA/PEG	0.968	2.54	0.960	0.017	0.971	17.7	0.932	0.458
CuO-NPs@L-PLGA/PDA/PEG	0.953	8.21	0.886	0.053	0.971	29.2	0.989	0.681

^(a)^Without laser irradiation; ^(b)^ Release exponent evaluated for n < 66% released

### 3.4. In vitro chemo-photothermal activity

**Figure 8.**
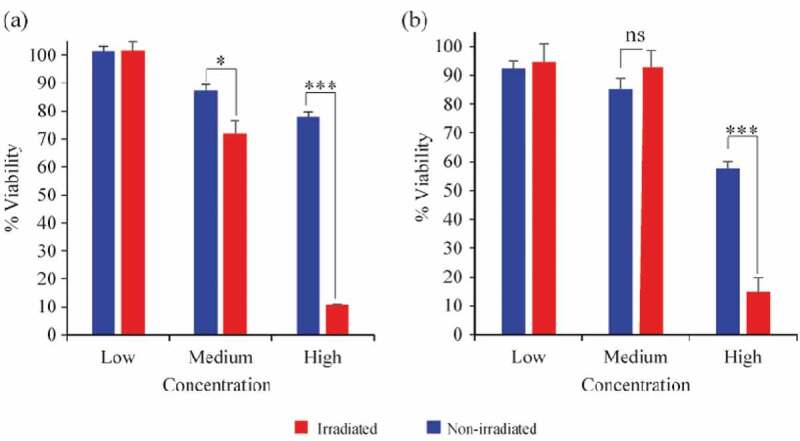
*In vitro* MTT cell viability assay of Cal-33 head and neck squamous cell carcinoma cells. Cells incubated with (a) CuO-NPs@H-PLGA/PDA/PEG or (b) CuO-NPs@L-PLGA/PDA/PEG at three concentrations (110, 55 and 27.5 μg Cu/mL). NIR laser Irradiated cells (red bars) non irradiated cells (blue bars). Experiments were done in triplicates and unpaired *t test* was performed to measure statistical significance. * P < 0.05, ***P < 0.001

The *invitro* cell viability assay showed that high concentrations (110 μg Cu/mL), both CuO-NPs@L-PLGA/PDA/PEG NS and CuO-NPs@K-PLGA/PDA/PEG NS have a strong photo-thermal effect on cell viability with more than 7 folds difference between irradiated and non-irradiated conditions (P < 0.001) as displayed in Figure 8. Interestingly, CuO-NPs@H-PLGA/PDA/PEG NS showed a significant photo-thermal effect even at medium concentration (55 μg Cu/mL) while CuO-NPs@L-PLGA/PDA/PEG NS coated did not (P < 0.05). These results indicate that the laser-induced thermal response and controlled release of the CuO-NPs lead to specific anti-cancer activity *invitro* at concentrations that are physiologically relevant.

Cal-33 is a PIK3CA driven cancer, which usually responds to PI3K inhibitors but with significant dose-limiting side effects of hyperglycemia and thus not yet approved to use for this indication [60]. Other compounds that are known to inhibit other head and neck cancer cells are platinum-containing drugs and radiotherapy [61], which also have dose-limiting side effects. Our approach can be used directly on tumors residing in the oral cavity which are accessible to laser-induced response. Further *invivo* experiments are warranted to evaluate its safety and efficacy profile *invivo*.

### 3.5. MRI

**Figure 9.**
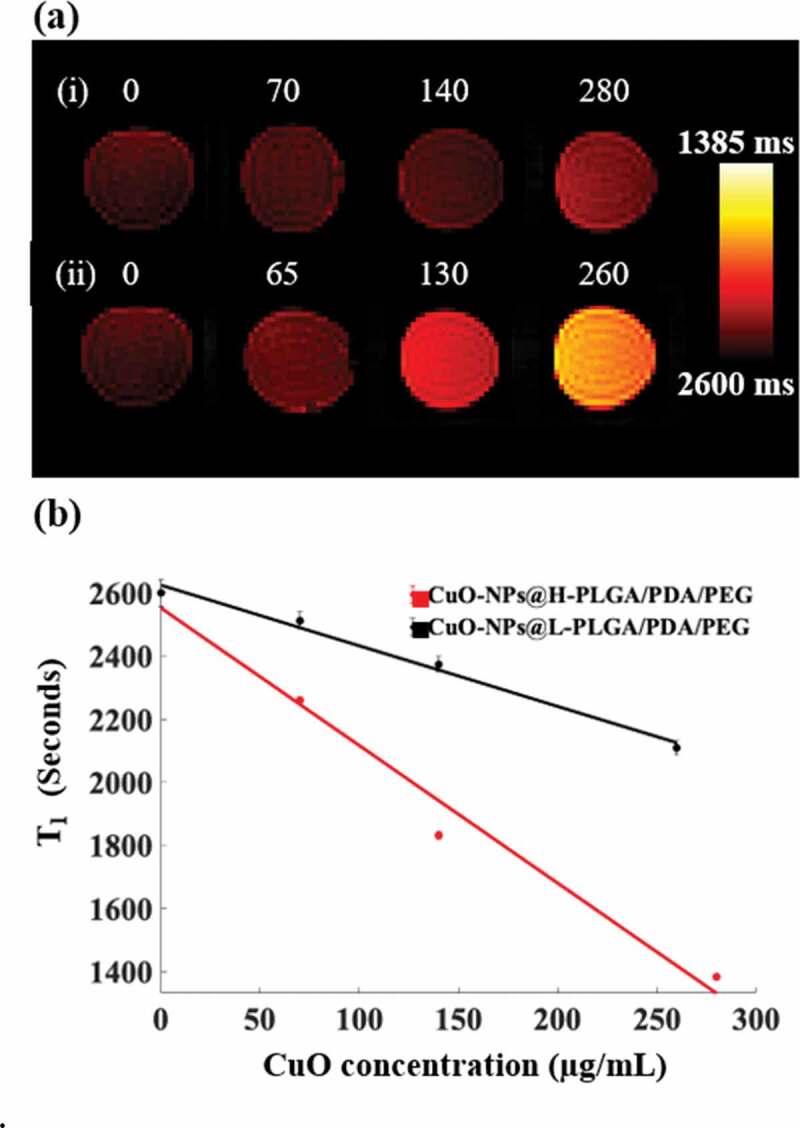
(a) MRI T1 mapping images of four test tubes containing solutions with different concentrations of CuO-NPs (µg/mL). (i) CuO-NPs@H-PLGA/PDA/PEG and (ii) CuO-NPs@L-PLGA/PDA/PEG. Note the substantially increased signal for the higher concentrations. (b) T1 values as a function of CuO-NPs concentration with the corresponding linear regression lines for both types of coating. Note the significant shortening of the T1 values for the H-PLGA/PDA/PEG formulation

The T1 mapping MRI scans revealed that the investigated CuO coatings generated augmented MRI contrast values as the CuO concentrations increased (Figure 9). This corroborates our previous finding [62] in which the ability of non-coated NS to serve as an MRI contrast enhancement material was described. Interestingly, the results obtained here indicate that the coating not only preserves this effect but also shows potential augmentation of the contrast. In quantitative terms, while for non-coated NS a signal increase of about 28% was achieved [62] at a CuO concentration of 200 μg/mL, here, the H-PLGA/PDA/PEG formulation achieved a contrast enhancement of approximately 36.5%. This further establishes the potential use of the suggested nano system for both diagnostic imaging and for therapy.

## 4. Conclusions

In summary, two delivery system formulations of CuO-NPs encapsulated in PLGA/PDA/PEG nanospheres, containing different polymeric MW of PLGA core, were synthesized and used to form stimuli-responsive multifunctional nanocarriers. The results show molecular weight-dependent, effective and rapid CuO-NPs release from PLGA/PDA/PEG carriers. These formulations exhibited heating efficiency always higher than 85% in comparison to uncoated CuO-NPs loaded with PLGA NS and water (control). The high heating efficiency with the associated controlled release, allows to design a thermal therapy approach which is capable of killing tumor cells with lower laser power and shorter time as compared to those required in conventional therapy. This can provide much-reduced collateral damage to healthy tissues surrounding the target.
